# Prevalence and types of medication errors in pro re nata medication orders among hospitalized patients: a cross-sectional study

**DOI:** 10.1186/s40780-025-00482-x

**Published:** 2025-08-09

**Authors:** Arefeh Rasouli-Rad, Mahdi Ahmadinia, Azadeh Eshraghi, Hamidreza Aslani, Zahra Karimian, Akram Hashemi, Maryam Farasatinasab

**Affiliations:** 1 Department of Clinical Pharmacy and Pharmacoeconomics, School of Pharmacy, Iran University of Medical Sciences, Tehran, Iran; 2 Lung Transplantation Research Center, National Research Institute of Tuberculosis and Lung Diseases (NRITLD), Shahid Beheshti University of Medical Sciences, Tehran, Iran; 3 International Affairs Office, Iran University of Medical Sciences (IUMS), Tehran, Iran; 4 Center for Educational Research in Medical Science (CERMS), Department of Medical Education, School of Medicine, Iran University of Medical Sciences, Tehran, Iran; 5 Medical Ethics Department, School of Medicine, Iran University of Medical Sciences, Tehran, Iran; 6 Firoozgar Clinical Research Development Center (FCRDC), Iran University of Medical Sciences, Tehran, Iran

**Keywords:** PRN prescriptions, Medication errors, Hospital safety, Prescribing practices, Patient safety

## Abstract

**Background:**

Medication errors in Pro Re Nata (PRN) prescriptions pose a significant threat in hospital settings, especially due to unclear prescribing practices. Despite growing attention to patient safety, documentation for PRN orders remains poor, increasing the risk of medication errors and adverse drug events. To assess the prevalence and types of PRN medication errors in hospitalized patients, identify high-risk drugs, and explore factors linked to prescribing errors.

**Methods:**

This cross-sectional study was conducted in 2023 at an Educational and Research Hospital. It included 400 hospitalized patients who had received at least one PRN prescription. Data were collected using a standardized extraction form based on clinical guidelines and expert consultation. Chi-square tests and logistic regression were used to evaluate error trends and associated risk factors.

**Results:**

A total of 74.1% of PRN prescriptions lacked a documented indication, and 91.1% had no recorded dosage interval. Pethidine (32.6%) was the most frequently prescribed PRN medication. The Surgical ICU showed a significantly higher number of errors (*p* < 0.05). Major predictors of PRN errors included missing dosage intervals and admission to high-dependency wards.

**Conclusions:**

The high frequency of PRN prescribing errors underscores the urgent need for improved documentation and targeted training. Structured interventions such as electronic prescribing and focused medical education can help reduce errors and improve patient safety. Structured interventions such as electronic prescribing, regulatory enforcement, and focused medical education can help reduce errors and improve patient safety.

**Supplementary Information:**

The online version contains supplementary material available at 10.1186/s40780-025-00482-x.

## Introduction

Medication errors are a significant global concern, contributing to approximately 10% of patient harms in healthcare settings. Notably, over 50% of this harm is preventable, with medications being a primary factor. In the United States, medical mistakes are acknowledged as a significant public health concern, with estimates indicating that more than 200,000 patient fatalities per year result from avoidable medical errors [1]. Among various categories of errors, PRN (pro re nata) medication orders, designed to be administered “as needed”, represent a high-risk area due to their inherent ambiguity in dosing, indication, timing, and documentation [2, 3].

Studies conducted in intensive care units (ICUs) have shown that PRN medications are responsible for 9–40% of all medication errors [4]. A prospective study in a large ICU in Saudi Arabia reported that 89% of PRN prescriptions contained at least one error, often related to incomplete documentation, incorrect dosages, or unclear frequency [5]. These findings are particularly concerning in settings lacking electronic prescribing systems, where errors may go unnoticed or uncorrected. In addition to ICU settings, PRN prescribing errors are also common in general hospital wards. For instance, up to 70% of PRN prescriptions lack a documented indication, and nearly 50% omit the dosing interval, contributing to polypharmacy, drug interactions, and unnecessary medication use [6, 7]. A 2019 study analyzing over 4 million outpatient prescriptions found that prescribers often neglect to record the rationale for drug use, leading to information gaps that compromise patient safety [8].

These kinds of mistakes are generally caused by a number of things, such as the lack of standardized institutional rules for PRN prescription, not getting enough training during medical school, and the tremendous workload and time pressure that physicians sometimes feel while working in hospitals [9]. These challenges are especially prominent in low- and middle-income countries (LMICs), where electronic health record (EHR) systems are often underdeveloped or unavailable [10]. The World Health Organization’s global patient safety challenge emphasizes medication safety improvements in LMICs, highlighting the disproportionate burden of preventable drug-related harm in these regions [11].

Prior literature on PRN prescribing has focused largely on psychiatric and long-term care settings in high-income countries [12]. A recent integrative systematic review of 31 studies on PRN medication practices revealed that most research has been concentrated in mental health care, with limited attention to general medical or surgical wards [13]. However, most of the available studies on PRN prescribing errors has focused on specialized settings such as ICUs and psychiatric wards, where medication regimens are often more complex. In contrast, data on PRN prescribing practices and associated errors in general medical and surgical wards remain scarce, despite the high volume of patients and frequent use of PRN medications in these environments [2]. This study specifically addresses this gap by evaluating PRN-related errors in general hospital wards, providing insights that are more applicable to routine inpatient care settings. Moreover, studies evaluating the effectiveness of interventions, such as automation, electronic systems, or prescriber training, on PRN medication safety are scarce [4, 12, 13].

One comparative study found that hospitals using EHR systems had significantly better documentation of PRN medication indications and outcomes compared to those using paper-based records [12]. Even though this research was done at a hospital that only uses paper-based prescription, samples from EHR systems are given to show how different they are and to stress how digital prescribing platforms might make PRN drug safety better. However, few investigations have examined how these technological differences impact medication safety in diverse clinical settings. In addition, the role of structured educational interventions in preventing PRN-related errors is often overlooked. Evidence suggests that incorporating pharmacovigilance and rational prescribing principles into internship and residency training can enhance clinical decision-making and reduce medication-related harm [6, 8].

This cross-sectional research looks at the incidence and kinds of medication errors that happen with PRN prescriptions in many hospital wards, such as internal medicine, surgery, the ICU, and burn units of a tertiary teaching hospital in Iran. It does this to fill in the gaps that are caused by many different factors. This setting, representing an LMIC with a non-electronic prescribing infrastructure, provides a unique opportunity to evaluate PRN-related risks in real-world practice. Medication errors in this study were defined according to the National Coordinating Council for Medication Error Reporting and Prevention (NCC MERP) as any preventable event that may cause or lead to inappropriate medication use or patient harm [14]. The findings aim to inform targeted improvements in clinical education, prescription protocols, and health system design—ultimately promoting safer use of PRN medications across diverse care environments.

## Materials and methods

### Study design and setting

A comprehensive cross-sectional study was conducted over more than six months during the year 2023 in Firoozgar Educational and Research Hospital, affiliated with Iran University of Medical Sciences. The principal objective of the study was to thoroughly investigate the various types and the prevalence of medication errors present in PRN prescriptions that had already been issued to hospitalized patients. This investigation was conducted across a wide range of hospital wards, including internal medicine wards, surgical wards, ICUs, and the burn unit, to enable a broad study of the issue in varied sections of the hospital. The study design follows the methodological framework commonly employed in research on medication safety and prescribing errors [8].

### Study population and sampling

The study population included all hospitalized patients who had at least one PRN medication order during their hospital stay. Stratified random sampling was employed to ensure that the data collected represented different hospital wards proportionally. Patients were eligible for inclusion if they were hospitalized for at least 24 h and had at least a single PRN medication order in their medical file.

Patients were excluded if their PRN medication orders lacked sufficient documentation to assess for errors, for example, if key prescribing information such as drug name or dose was entirely missing from the order sheet, making evaluation impossible. In contrast, patients whose PRN orders contained incomplete elements (e.g., missing indication or frequency) were included and classified as having documentation errors according to the study objectives. Pediatric patients (under 18 years of age) were excluded due to distinct prescribing protocols and dosing standards that differ significantly from adult populations, which were beyond the scope of this study. Patients admitted to palliative care units were also excluded because PRN prescribing practices in these settings are tailored to end-of-life comfort care needs and follow different documentation standards, which could introduce bias when comparing with general medical and surgical wards.

The required sample size was estimated using the standard single-proportion formula for cross-sectional studies: n = (Z² × P × (1 − P)) / d², where Z is the standard normal deviate at 95% confidence level (1.96), P is the estimated proportion of PRN prescribing errors (assumed to be 0.20 based on previous literature), and d is the margin of error (0.04). Based on this calculation, a sample size of 384 prescriptions was determined to be sufficient. To account for potential data loss and ensure analytical power, the sample size was increased to 400 prescriptions. This approach is consistent with similar medication error studies in inpatient settings [15].

### Data collection and variables

The collection of data was carried out through a structured extraction form that was prepared according to clinical guidelines, previous literature, and expert consultations. The extraction form covered several variables related to patient demographics, prescribing patterns for medications, and quality of prescription documentation. Demographic details such as patient’s age, gender, particular diagnosis, explanation for their hospitalization, and other comorbidities were documented as the purpose of describing patient features.

The PRN prescription was thoroughly examined, encompassing the medication name, therapeutic class, indication for use, prescribed dose, route of administration, frequency of administration, and maximum daily allowable dose. The assessment included documentation and prescribing errors, emphasizing missing indications, prescribing details, expiration, recognized clinical prescribing standards, and the use of non-preferred abbreviations. To ensure consistent and accurate identification of medication errors, all PRN orders were to be cross-referenced with the guidelines established by Lexicomp^®^ and the national pharmaceutical protocol [14]. Any departure from established prescribing practices was categorized as a potential medication error.

### Medication error assessment

PRN prescriptions were reviewed by two independent clinical pharmacists using a standardized error identification checklist derived from institutional protocols and Lexicomp^®^ references. The checklist included key elements such as indication, dose, route, frequency, dosage interval, and duration. Any omission or deviation from standard prescribing criteria was classified as a medication error. Disagreements between reviewers were resolved through consensus discussion, or, if needed, by consulting a third senior pharmacist. Errors were categorized as documentation errors (missing information), prescribing errors (incorrect entries), or abbreviation-related issues.

In this study, medication errors were categorized into three groups for clarity and consistency. Documentation errors referred to omissions of essential information, such as indication, frequency, or duration, that could make the order unclear or incomplete for administration. Prescribing errors were defined as inaccurate entries within the PRN order, such as incorrect dose, route, or frequency, when compared with Lexicomp or institutional guidelines. Abbreviation-related issues refer to the use of ambiguous or non-standard abbreviations that might lead to misinterpretation by healthcare staff.

In this study, prescribing errors were defined specifically as documentation-related and prescribing entry errors within PRN orders. Broader categories of prescribing errors, such as inappropriate drug selection, overlooked contraindications, and potential drug–drug interactions, were not included in the evaluation due to the need for comprehensive clinical appropriateness assessments requiring full chart review and physician input, which were beyond the scope and ethical approvals of this project.

Examples of incorrect entries assessed included cases where the prescribed dose exceeded the maximum recommended PRN dose according to Lexicomp guidelines (e.g., pethidine prescribed above 100 mg per administration) or where the administration route was incorrectly documented (e.g., prescribing a medication approved only for IV use as IM).

### Statistical analysis

All statistical analyses were conducted using SPSS version 2022 (IBM Corp., Armonk, NY). Descriptive statistics, including means, standard deviations, and frequency distributions, were used to summarize demographic data and prescription trends. The chi-square test was applied to examine the association between PRN prescription errors and hospital wards. Normality of continuous variables analyzed by ANOVA was assessed using the Shapiro-Wilk test, and assumptions for parametric analysis were met. One-way ANOVA was performed to compare the mean number of medication errors across different hospital wards.

For the multivariable logistic regression analysis, we pre-specified a core set of clinically and theoretically relevant predictors a priori (admission to the Surgical ICU, absence of indication specification, lack of minimum interval documentation, and lack of documented dosage) based on existing literature and clinical plausibility [16, 17]. In addition, candidate exploratory variables were considered for inclusion if they showed an association with PRN prescription errors at *p* < 0.20 in the univariate analysis [18, 19]. Stepwise selection procedures were deliberately avoided due to their known limitations, including model instability and biased parameter estimates [20]. Exploratory variables considered in earlier drafts (e.g., prescriber level and dosing frequency) were not retained in the final model because they were not consistently available and did not meet our inclusion criteria. To adjust for potential confounding, age group, gender, and the presence of major comorbidities (hypertension, diabetes, and cancer) were included regardless of their univariate significance because these variables are well-established confounders of medication safety outcomes [16, 17]. Age group was modeled as three categories (18–49, 50–64, and ≥ 65 years) using two dummy variables, and comorbidities were entered as separate binary covariates rather than as a composite variable. This structure resulted in a total of 10 predictor parameters in the final model. Multicollinearity among independent variables was assessed using the Variance Inflation Factor (VIF), applying a conservative threshold of VIF < 5; all included variables showed VIF values below 2, indicating no significant collinearity concerns. Linearity of the logit for continuous variables was evaluated using the Box–Tidwell approach, and no meaningful violations were observed [21]. The total number of PRN prescription error events and the number of predictors were used to calculate the events-per-variable (EPV) ratio, which exceeded the conventional threshold of 10 [18, 21]. Model fit and discrimination were assessed using the Hosmer–Lemeshow goodness-of-fit test, the area under the receiver operating characteristic curve (AUC), and calibration intercept and slope. In addition, a pre-specified sensitivity analysis was conducted to examine the robustness of the model by excluding the variable “lack of documented dosage” (*n* = 6) because of its small event count [21]. Results of the regression model are presented as both unadjusted and adjusted odds ratios (ORs) with 95% confidence intervals (CIs).

For visual clarity, pie charts were developed for missing prescription indications, bar charts for PRN prescription trends, and a forest plot was created to illustrate adjusted odds ratios. Additionally, the relationship between patient age and PRN prescription errors was shown in a supplementary figure.

## Results

### Demographic characteristics and patient profiles

A total of 400 patients were included in this study, of whom 248 (62%) were male and 152 (38%) were female. The mean age of the patients was 53 ± 18.06 years. The most common reasons for hospitalization were fractures (19%), cancer (18.7%), and intracranial hemorrhage (2.5%). Regarding underlying comorbidities, hypertension (21.5%), previous surgery (14.5%), and diabetes (10.6%) were the most prevalent conditions (Table 1).

**Table 1:** Demographic characteristics and comorbidities of hospitalized patients. Data are presented as frequency (percentage) or mean ± SD

Category	Count	Percentage	MD	SMD
Patient Demographics				
Male	248	62%	2.5	0.12
Female	152	38%	2.1	0.1
Causes of Hospitalization				
Fracture	76	19%	4.1	0.18
Cancer	75	18.70%	3.9	0.17
Subarachnoid Hemorrhage	30	7.50%	1.5	0.07
Multiple Trauma	28	7%	1.3	0.06
Abdominal Pain	25	5%	1.2	0.05
Decreased Consciousness	14	3.50%	0.7	0.03
Ischemic Stroke	14	3.50%	0.6	0.03
Sepsis	12	3%	0.5	0.02
Bariatric Surgery	10	2.50%	0.4	0.02
Intracranial Hemorrhage	10	2.50%	0.3	0.01
Gastrointestinal Bleeding	7	1.80%	0.2	0.01

Abbreviations: MD = Mean Difference (compared to overall patient mean); SMD = Standardized Mean Difference. MD represents the absolute difference from the overall mean value for each category, and SMD indicates the effect size standardized by the pooled standard deviation. These measures illustrate the magnitude of variation in demographic and clinical characteristics within patient subgroups

Note: Cause-of-hospitalization data were available and classifiable for 301 patients. The remaining cases had missing, non-specific, or uncategorizable admission diagnoses

### PRN prescription patterns in the hospital

**Fig. 1:**
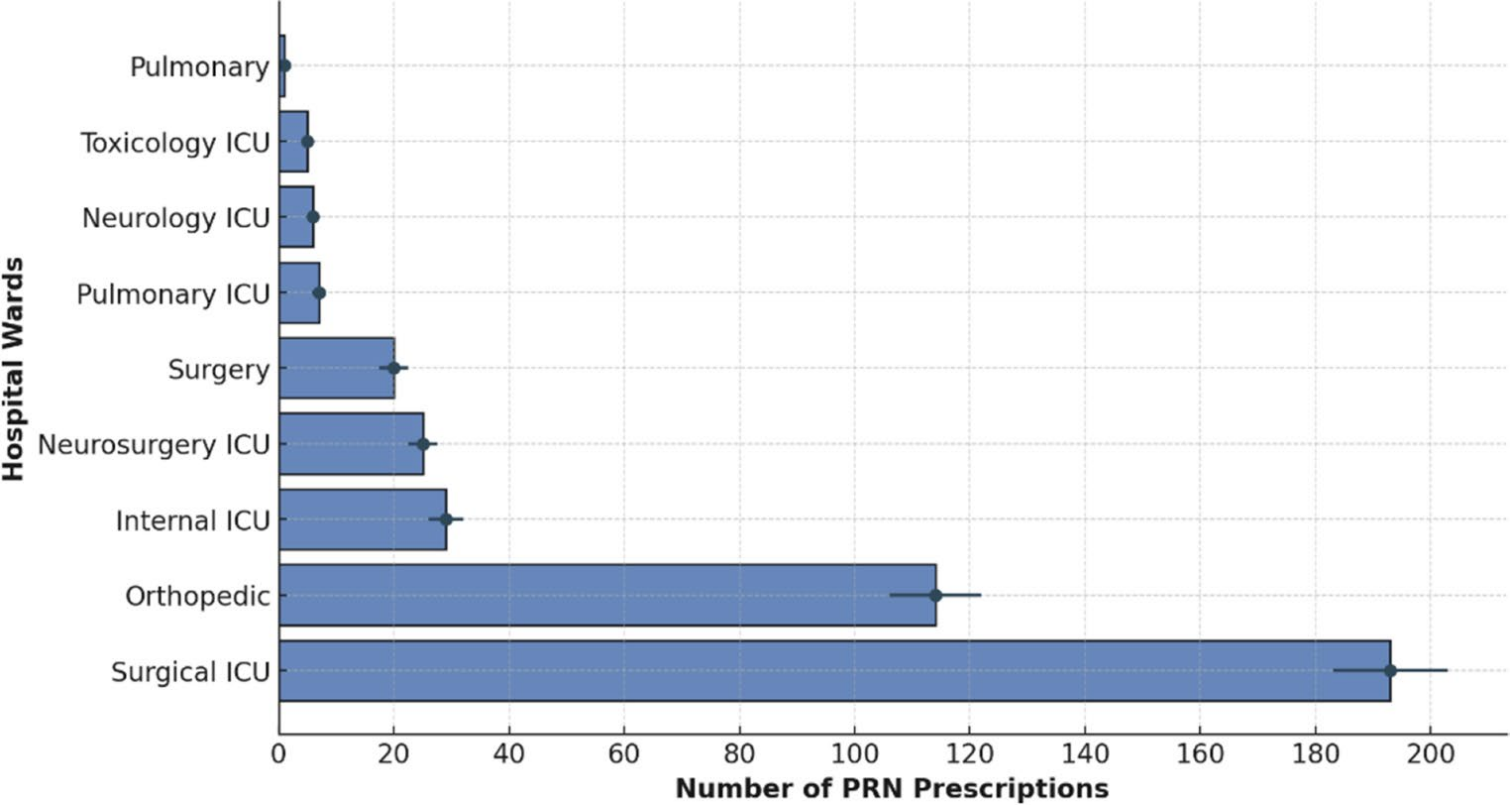
PRN prescription distribution across hospital wards. Blue bars indicate prescription counts, black lines represent standard errors

An analysis of PRN prescription distribution across hospital wards was conducted based on data from 389 patients with clearly documented ward assignments. The highest number of PRN prescriptions was recorded in the surgical ICU (*n* = 193; 48.25%) and the orthopedic ward (*n* = 102; 25.5%). Moderate frequencies were observed in the internal ICU (*n* = 29; 7.25%) and neurosurgery ICU (*n* = 23; 5.75%), followed by the surgical ward (*n* = 20; 5.0%). The pulmonary ICU (*n* = 8; 2.0%), neurology ICU (*n* = 7; 1.75%), toxicology ICU (*n* = 6; 1.5%), and the pulmonary ward (*n* = 1; 0.25%) had the lowest proportions. Data from 11 patients were excluded from this analysis due to missing or non-standard ward documentation. The complete distribution is illustrated in Fig. 1.

Pethidine was the most frequently prescribed PRN medication, while fentanyl, metoclopramide, and diazepam were among the least used. Notably, a large proportion of PRN prescriptions lacked a documented indication. A comprehensive breakdown of PRN drugs and their documentation quality is provided in Supplementary Table 1S; MD values were calculated to quantify differences in prescribing frequency across medications.

### Quality of documentation and PRN prescription errors

Types of PRN-related errors were categorized and their frequencies were calculated based on predefined criteria. Documentation errors were the most common, particularly missing indications and dosage intervals. The breakdown of error types is shown in Table 2s.

Analysis of prescription documentation quality revealed that 94.7% of PRN orders contained no abbreviations. However, only 8.9% of prescriptions included a specified minimum interval between doses, and none of the prescriptions documented the duration of PRN medication use (Table 2). These findings highlight significant gaps in documentation practices related to PRN prescriptions. Further insights into the relationship between underlying comorbidities and PRN prescription errors are presented in the supplementary material (Table 3S). Documentation and prescribing errors were identified using predefined criteria based on hospital protocols and Lexicomp standards.

**Table 2:** Documentation quality of PRN prescriptions. Includes use of abbreviations, dosage intervals, and duration of administration

Documentation Quality	Yes (%)	No (%)
Use of Abbreviations	21 (5.3%)	380 (94.7%)
Indication Documented	104 (25.9%)	297 (74.1%)
Dosage Documented	395 (98.5%)	6 (1.5%)
Minimum Interval Documented	36 (8.9%)	365 (91.1%)
Duration Documented	0 (0%)	401 (100%)

### Association between hospital wards and PRN prescription errors

**Fig. 2:**
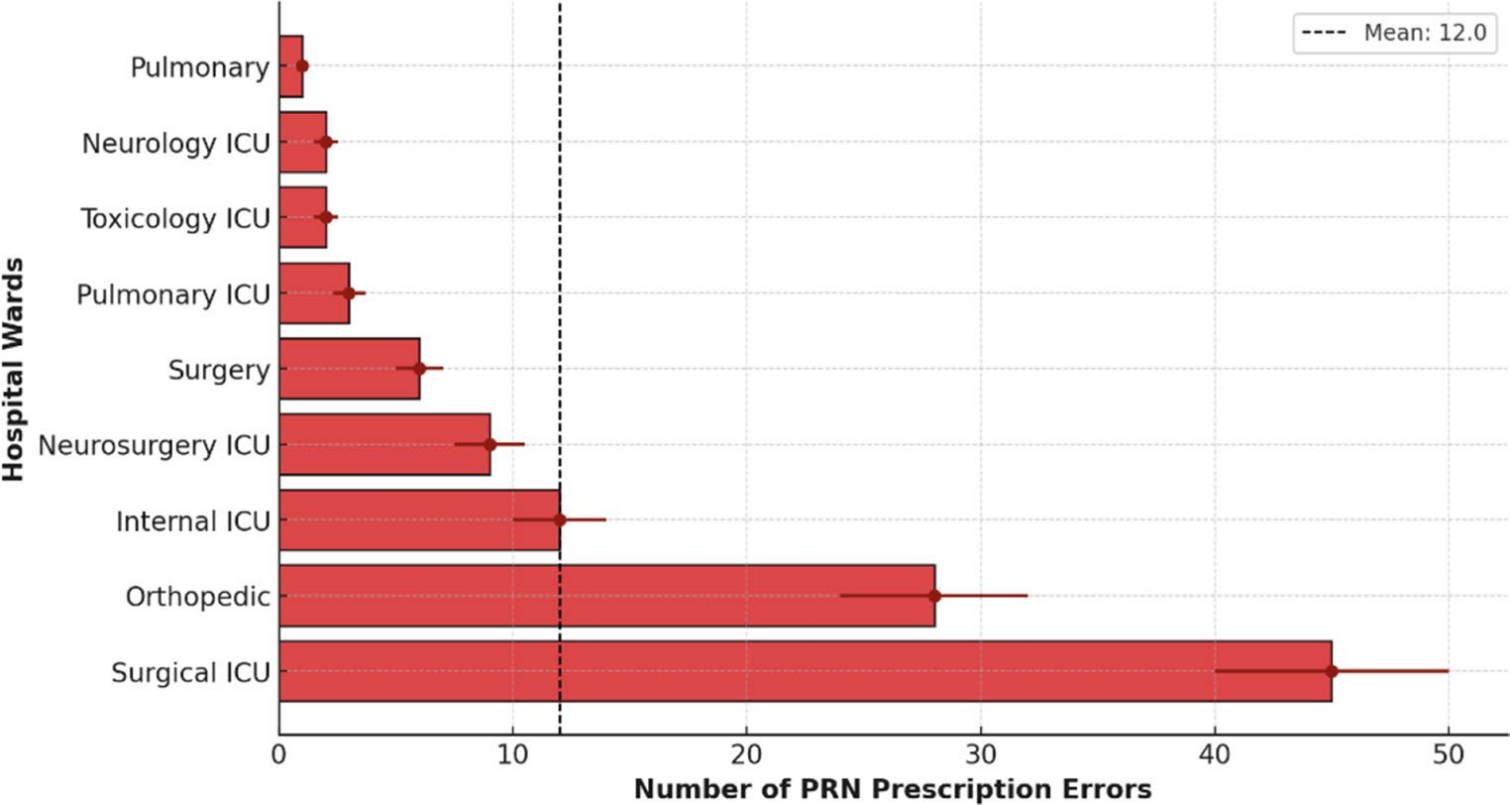
Comparison of PRN prescription errors across hospital wards. The dashed line shows the average error rate

In the initial univariate analysis using chi-square tests, we explored the distribution of PRN prescription errors across hospital wards and selected patient subgroups. A comparison of prescription errors across hospital wards showed that medication errors were significantly higher in Surgical ICU compared to other units (*p*-value < 0.05) (Fig. 2). Additionally, a significant association was observed between the type of disease and the likelihood of PRN prescription errors, with cancer and multiple trauma patients experiencing the highest error rates (χ² = 12.3, *p* = 0.004). A supplementary analysis of the correlation between patient age and PRN prescription errors is presented in Fig. 1S.

### Factors associated with PRN prescription errors

**Fig. 3:**
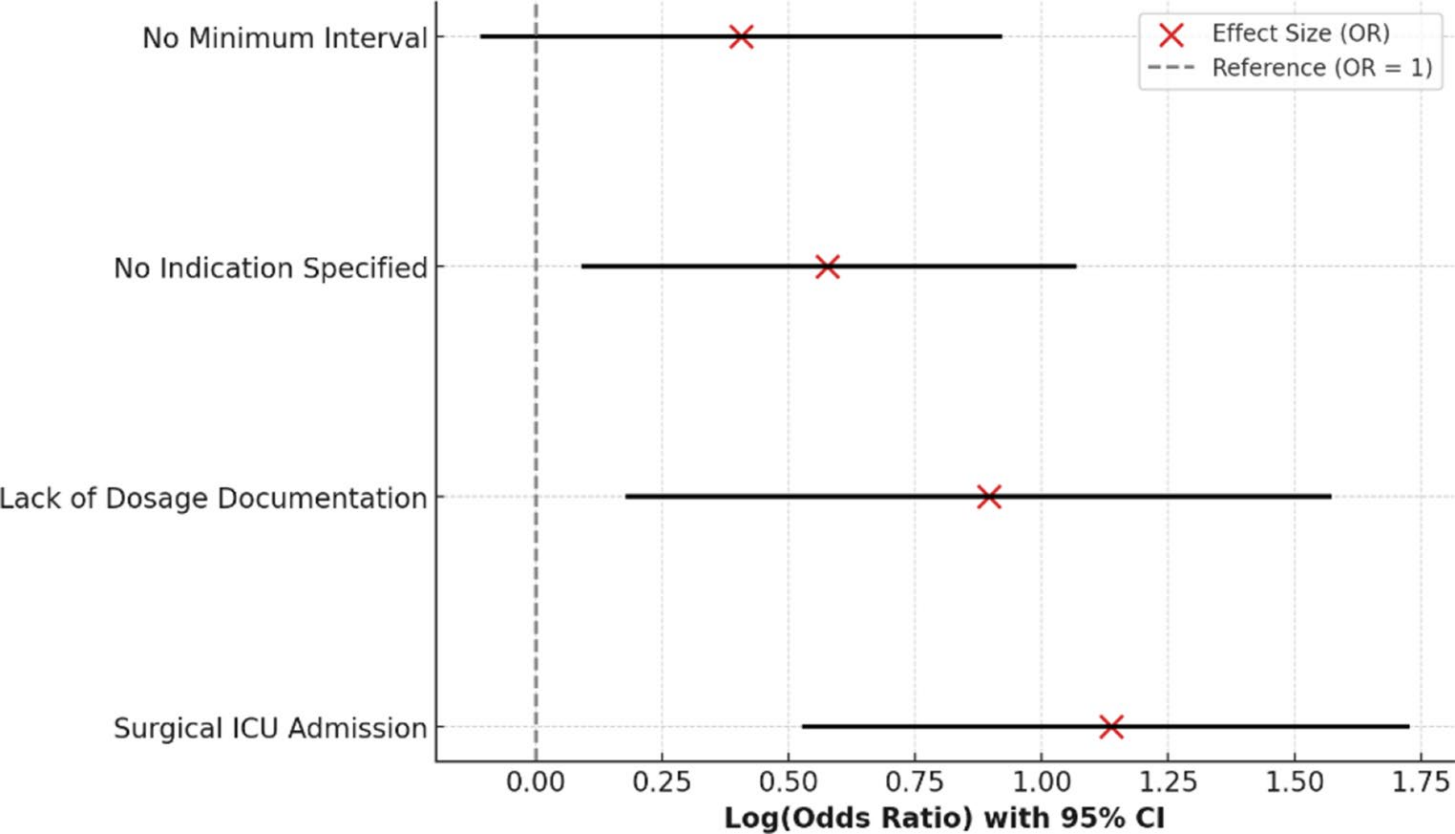
Factors associated with PRN prescription errors. Odds ratios (ORs) and 95% confidence intervals are presented in a forest plot. The dashed line indicates reference (OR = 1). Note: ORs derived from multivariable logistic regression adjusted for age, gender, and comorbidities (hypertension, diabetes, cancer)

In the regression sample comprising 389 patients with complete covariate data, there were 349 events and 40 non-events at the patient level. With 10 predictor parameters in the final model, the events-per-variable (EPV) ratio was 34.900, substantially exceeding the conventional threshold of 10 and indicating a low risk of model overfitting. Because the regression analyses were performed at the patient level (*n* = 389) with the outcome defined as the presence of any PRN prescription error per patient, the total number of observations differs from the counts reported in Supplementary Table 1, which are presented at the prescription (order) level (*n* = 401) by design. To identify independent predictors of PRN prescription errors, a multivariable logistic regression model was constructed, adjusting for key demographic and clinical variables. The model included the following predictors: admission to the Surgical ICU, lack of documented dosage, absence of documented indication, and lack of minimum interval specification. Confounding variables such as patient age group, gender, and presence of comorbidities (hypertension, diabetes, and cancer) were also included to adjust for potential bias. The final multivariable logistic regression model demonstrated good calibration (Hosmer–Lemeshow χ² = 8.137, df = 8, *p* = 0.429) and acceptable discrimination (AUC = 0.781, 95% CI: 0.701–0.848). Calibration intercept and slope were also examined and supported the adequacy of the model fit. To further assess the robustness of the findings, we conducted a sensitivity analysis excluding the variable “lack of documented dosage” (*n* = 6) because of its small event count. The results were materially unchanged: the discrimination (AUC = 0.777, 95% CI: 0.699–0.846) and calibration (Hosmer–Lemeshow *p* = 0.412) remained comparable to the full model, and the odds ratios for the remaining predictors changed by less than 1%. The results indicated that admission to the Surgical ICU (OR = 3.12, 95% CI: 1.7–5.6, *p* < 0.01) and lack of documented dosage (OR = 2.45, 95% CI: 1.2–4.8, *p* = 0.02) were the strongest predictors of PRN medication errors (Fig. 3).

## Discussion

The findings of this study revealed a high prevalence of documentation deficits in PRN medication orders, namely in the absence of indications, dosing frequency, and duration of administration. This is an indication of the need for increased monitoring in PRN prescribing practices. This is in line with previous research that indicates PRN medication mistakes are common within hospital settings, primarily due to non-adherence to documentation norms [9]. Prior work in this area has also described similar documentation deficits in a range of healthcare settings, as indeed is the situation with such a high rate of missing indications in 74.1% of PRN prescriptions in the current study [3]. A comparison of the findings of these studies with prior work indicates that orthopedic wards and surgical intensive care units experience higher rates of PRN prescription errors than other hospital departments. According to existing research, patients on such high-dependency wards are at higher risk of drug errors because of the complexity of their treatment regimens and the higher chance that they will need PRN medication for sedation and pain [22, 23]. In this paper, the authors also found an association between a higher risk of PRN prescription error and some comorbidities, such as multiple trauma and cancer. Studies indicate that patients with complex medical conditions, particularly those requiring polypharmacy, are at higher risk of medication-related errors [24, 25]. Furthermore, the small SMD values calculated for demographic and comorbidity variables indicate minimal effect sizes, suggesting that age, gender, and common comorbidities were relatively evenly distributed in the study population and were unlikely to confound the observed PRN prescribing errors.

Surgical ICU admission being linked with a threefold higher odds of PRN prescribing errors is consistent with the literature on ICU medication safety. Critically ill patients often require complex, rapidly changing drug regimens, and intensive care settings are known to have high medication error rates under heavy workload and time [26, 27]. Indeed, a recent review identified ICU-specific risk factors– including high illness severity, staff overload and frequent interruptions– that contribute to prescribing [26]. Encouragingly, these risks are modifiable. Interventions such as pharmacist-led order review and structured checklist-based prescribing in ICUs have shown efficacy in catching errors early and reducing adverse drug events [26]. The strong association between undocumented dosage and errors further underscores the importance of complete orders. Incomplete prescriptions are a well-documented root cause of medication errors in critical care, accounting for roughly half of observed prescribing errors in one ICU [28]. The omission of a dosage, a critical parameter, may result in delays or inconsistencies in administration, necessitating that nurses interpret or clarify the order. This error type is preventable. Implementing standardized prescription templates and mandatory fields in electronic prescribing systems ensures that no dose is unspecified. Additionally, staff training in comprehensive medication documentation is recommended to enhance compliance [26, 27].

With respect to patient age, our analysis indicated that older adults (≥ 65 years) experienced higher PRN error rates. This trend aligns with broader findings that geriatric patients are especially vulnerable to medication errors due to polypharmacy and complex comorbidities [29]. Notably, one recent study reported that patients over 75 faced a 38% higher risk of medication errors, and those ≥ 65 had nearly double the medication-related hospital admissions of younger patients [29]. Pharmacokinetic alterations associated with aging and cognitive difficulties may additionally influence this vulnerability. These observations underscore the necessity for heightened vigilance and customized safety protocols, such as targeted medication reviews or consultations with geriatric practicioners, when prescribing PRN medications for elderly patients.

A medical explanation of the same identifies high workload among doctors, particularly acute ward doctors of hospitals, as one of the principal causes of PRN drug error. Literature has established that higher work pressure and staffing shortages directly influence PRN prescribing quality and, in most cases, lead to missing information on prescriptions [30]. The lack of standardized ordering procedures for PRN medications on some hospital wards could be an additional contributing factor. It is proven in previous studies that utilization of structured interventions, such as staff training programs and standardized checklists, can significantly restrict prescribing errors [31, 32].

The present study also indicates significant gaps in documentation of PRN prescriptions. Interestingly, minimum intervals between doses were mentioned in as low as 8.9% of the prescriptions, and none documented the duration of use of PRN medication. The lack of documentation may lead to patients being administered too much dose, thereby elevating the risk of adverse drug reactions. Studies have indicated that insufficient documentation of the dosing intervals of PRN drugs has been associated with medication overuse, potential for dependence, and a higher incidence of side effects, particularly of sedatives and analgesic groups [33, 34].

One of the strengths of this study is its cross-sectional design, conducted in a reputable teaching and research hospital, which allowed thorough examination of PRN medication errors across different hospital departments. In addition, the use of standardized prescription review criteria assisted in raising the validity of the findings. The relatively large sample size of 400 patients also adds strength to the study, since the findings are more representative than those of some previous studies with smaller sample sizes [35].

However, there are some limitations of this study. One such major limitation is that it failed to quantify the clinical consequences of PRN medication errors. Some studies have found that inadequate documentation of PRN prescriptions leads to increased hospital readmission and an increased number of drug-related adverse events [4, 36]. Future studies need to quantify the direct impact of PRN prescription errors on patient outcomes. Also, because this study was conducted in one hospital alone, how much the results can be extrapolated to other health facilities can be limited [37].

With these findings on hand, proper correction of errors in PRN prescriptions requires an integrated approach entailing better prescribing practices and intensified regulatory practices. Recent studies have emphasized that including electronic prescribing tools and real-time monitoring of the medication can prove to be significant in reducing the incidence of errors in PRNs [38]. Moreover, reinforcing the sensitization of medical personnel regarding the importance of correct documentation of PRNs and imposing severe institutional regulations on medication safety has been put forth as a viable measure [39].

These findings have the important implication that PRN medication safety should be incorporated into medical education curricula. The curriculum should include real-world case studies and epidemiologic data from studies like this one to prepare medical students, interns, and residents to better recognize and prevent PRN-related prescribing errors. Targeted educational engagement has been proven effective in this regard, with several studies noting the effectiveness of doing so in reducing medication errors and improving prescribing practices among trainees [33].

This research was performed in a singular tertiary teaching hospital in Iran, which may restrict the applicability of its results to comparable healthcare environments, especially in low- and middle-income countries lacking electronic prescribing systems. Variations in national regulations, institutional policies, and healthcare infrastructures among countries may affect PRN prescribing practices and the incidence of medication errors. This study has a limitation in that it did not evaluate broader categories of prescribing errors, including inappropriate drug selection, overlooked contraindications, or potential drug–drug interactions. Assessing these factors necessitates thorough clinical judgment, complete chart reviews, and direct physician evaluations, which exceeded the scope and ethical approvals of the present study. Future research that includes these dimensions will enhance the understanding of PRN medication safety in hospital environments.

## Conclusion

This study emphasizes the severity of the issue of PRN prescription errors and the urgency for systematic interventions to maximize medication safety. The frequent incomplete documentation, particularly the absence of indication and dosage interval, suggests the need for more refined prescribing guidelines. Closing these gaps requires a combination of structured training modules, standard prescribing guidelines, and technological advances such as electronic prescription monitoring. By implementing these practices, hospitals can significantly reduce medication errors, foster patient safety, and improve overall health outcomes. Future research should try to determine the clinical importance of PRN medication errors and create new strategies for preventing these dangers in diverse healthcare settings. In addition to targeted education and improved documentation, establishing stricter institutional regulations and enhancing national medical laws are also essential components to effectively reduce PRN prescribing errors and ensure patient safety.

## Electronic supplementary material

Below is the link to the electronic supplementary material.

Supplementary Material 1

## Data Availability

The datasets generated and/or analyzed during the current study are available from the corresponding author upon reasonable request.

## Abbreviations

PRN: Pro Re Nata
ICU: Intensive Care Unit
EHR: Electronic Health Record
LMICs: Low- and Middle-Income Countries
NCC MERP: National Coordinating Council for Medication Error Reporting and Prevention
OR: Odds Ratio
CI: Confidence Interval
SD: Standard Deviation
MD: Mean Difference
SMD: Standardized Mean Difference
SPSS: Statistical Package for the Social Sciences
