# Supplementary material for: Prevalence and types of medication errors in pro re nata medication orders among hospitalized patients: a cross-sectional study
Source: J Pharm Health Care Sci. 2025 Aug 9;11:70. doi: 10.1186/s40780-025-00482-x (PMC12681110; doi:10.1186/s40780-025-00482-x)
Supplement: Supplementary file 1 — Supplementary Material 1 [file 40780_2025_482_MOESM1_ESM.docx]

*Table Supplementary 1. Types of PRN medications and their documentation quality. Includes dosage forms, frequency, indications, and variability indicators (MD).*

| **Medication** | **Form** | **Frequency** | **Percentage** | **Indication** | **MD** |
| --- | --- | --- | --- | --- | --- |
| **Pethidine** | Ampoule | 129 | 32.60% | Moderate to Severe Pain | 3.2 |
| **Ondansetron** | Ampoule | 64 | 16.20% | Nausea and Vomiting | 2.5 |
| **Acetaminophen** | Ampoule | 63 | 15.90% | Fever or Pain | 2.1 |
| **Labetalol** | Ampoule | 32 | 8.10% | Blood Pressure > 160/100 mmHg | 1.8 |
| **Midazolam** | Ampoule | 27 | 6.80% | Sedation and Anxiety | 1.6 |
| **Morphine** | Ampoule | 21 | 5.30% | Severe Pain | 1.3 |
| **Ketorolac** | Ampoule | 17 | 4.30% | Acute Pain Management | 1.2 |
| **Fentanyl** | Ampoule | 7 | 1.80% | Postoperative Pain | 0.9 |
| **Metoclopramide** | Ampoule | 6 | 1.50% | Nausea and Vomiting | 0.7 |
| **Biperiden** | Ampoule | 6 | 1.50% | Extrapyramidal Symptoms | 0.6 |
| **Haloperidol** | Ampoule | 5 | 1.30% | Agitation and Psychosis | 0.5 |
| **Naloxone** | Ampoule | 3 | 0.80% | Opioid Overdose | 0.4 |
| **Methadone** | Ampoule | 3 | 0.80% | Chronic Pain, Opioid Dependence | 0.4 |
| **Diclofenac** | Suppository | 3 | 0.80% | Inflammatory Pain | 0.3 |
| **Diazepam** | Ampoule | 2 | 0.50% | Seizure, Muscle Spasm | 0.3 |
| **Atropine** | Ampoule | 2 | 0.50% | Bradycardia | 0.2 |
| **Dextrose 50%** | Vial | 1 | 0.30% | Blood Glucose < 70 mg/dL | 0.2 |
| **Digoxin** | Ampoule | 1 | 0.30% | Atrial Fibrillation, Heart Failure | 0.2 |
| **Glycerin** | Suppository | 1 | 0.30% | Constipation | 0.2 |
| **Lactulose** | Syrup | 1 | 0.30% | Constipation, Hepatic Encephalopathy | 0.1 |
| **Midodrine** | Tablet | 1 | 0.30% | Hypotension | 0.1 |
| **Alprazolam** | Tablet | 1 | 0.30% | Anxiety, Panic Disorder | 0.1 |
| **Opium** | Oral | 1 | 0.30% | Chronic Pain | 0.1 |
| **Promethazine** | Ampoule | 1 | 0.30% | Allergy, Motion Sickness | 0.1 |
| **Captopril** | Tablet | 1 | 0.30% | Blood Pressure > 160/100 mmHg | 0.1 |
| **Oxycodone** | Tablet | 1 | 0.30% | Moderate to Severe Pain | 0.1 |
| **Valsartan/Amlodipine** | Tablet | 1 | 0.30% | Blood Pressure > 140/90 mmHg | 0.1 |

*Abbreviations: MD = Mean Difference; “Mean” refers to the average number of PRN-related prescription errors identified per medication order for each drug.*

*Note: The total number of PRN prescriptions (n = 401) exceeds the number of patients (n = 400) because one patient received two separate PRN medication orders.*

*Table Supplementary 2. Classification of PRN medication errors. Categories include documentation errors, prescribing errors, and abbreviation-related errors.*

| Type of PRN Error | Frequency (n) | Percentage (%) |
| --- | --- | --- |
| **Missing indication** | 297 | 74.1 |
| **Missing dosage interval** | 365 | 91.1 |
| **No documented duration** | 401 | 100.0 |
| **Prescribing errors** | 11 | 2.8 |
| \| **Use of non-standard abbreviations** \| \| --- \|  \|  \| \| --- \| | 21 | 5.3 |

*Note: Prescribing errors were defined as incorrect entries, which in this study included incorrect dose or route.*

*Table Supplementary 3. Association between comorbidities and PRN prescription errors. The highest error rates were observed in patients with chronic kidney disease and neurological disorders.*

| **Comorbidity** | **Total Patients (N)** | **Patients with PRN Errors (N, %)** |
| --- | --- | --- |
| **Hypertension** | ***107*** | ***56 (52.3%)*** |
| **Diabetes** | ***53*** | ***29 (54.7%)*** |
| **Cancer** | ***75*** | ***44 (58.7%)*** |
| **Chronic Kidney Disease** | ***6*** | ***5 (83.3%)*** |
| **Neurological Disorders** | ***15*** | ***10 (66.7%)*** |
| **Respiratory Disease** | ***4*** | ***3 (75.0%)*** |

**
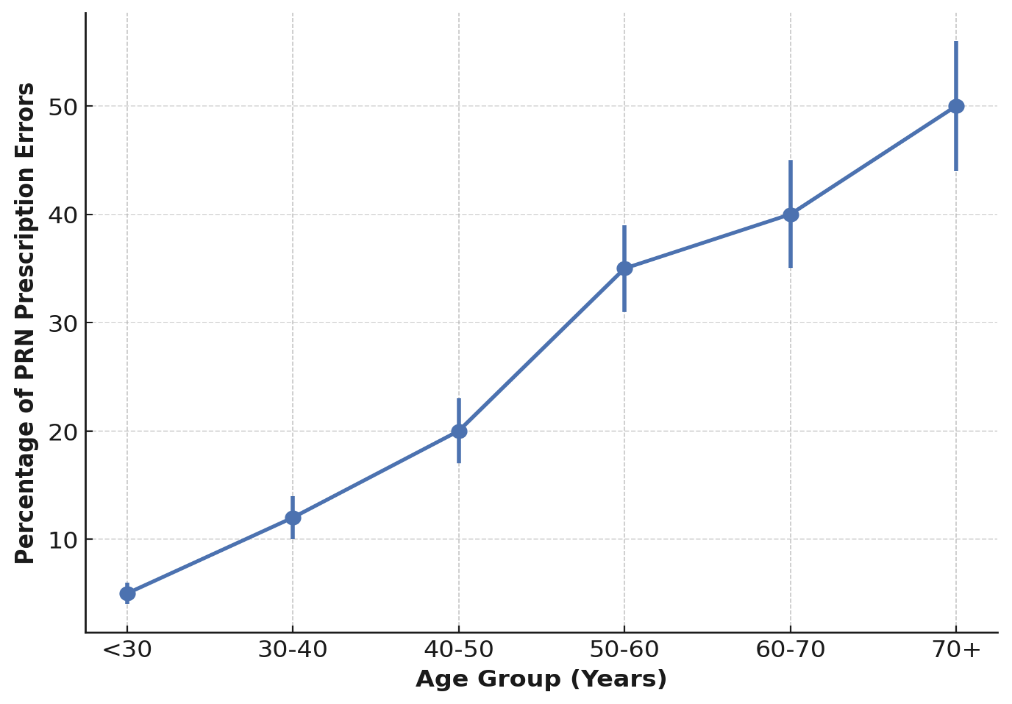
**

*Figure Supplementary 1. PRN prescription errors by age group. Error rates are stratified by age; error bars represent standard errors.*
